# CXCR2 signaling regulates *KRAS^(G12D)^*-induced autocrine growth of pancreatic cancer

**DOI:** 10.18632/oncotarget.6906

**Published:** 2016-01-13

**Authors:** Abhilasha Purohit, Michelle Varney, Satyanarayana Rachagani, Michel M. Ouellette, Surinder K. Batra, Rakesh K. Singh

**Affiliations:** ^1^ Department of Pathology and Microbiology, 985900 Nebraska Medical Center, Omaha, NE, USA; ^2^ Department of Biochemistry and Molecular Biology, 985870 Nebraska Medical Center, Omaha, NE, USA; ^3^ Eppley Institute, 985950 Nebraska Medical Center, Omaha, NE, USA

**Keywords:** KRAS^(G12D)^, PDAC, CXCR2, autocrine growth, ERK

## Abstract

Pharmacological inhibition of *RAS*, the master regulator of pancreatic ductal adenocarcinoma (PDAC), continues to be a challenge. Mutations in various isoforms of *RAS* gene, including *KRAS* are known to upregulate CXC chemokines; however, their precise role in KRAS-driven pancreatic cancer remains unclear. In this report, we reveal a previously unidentified tumor cell-autonomous role of *KRAS^(G12D)^*-induced CXCR2 signaling in mediating growth of neoplastic PDAC cells. Progressively increasing expression of mCXCR2 and its ligands was detected in the malignant ductal cells of Pdx1-cre;LSL-*Kras^(G12D)^* mice. Knocking-down CXCR2 in *KRAS^(G12D)^*-bearing human pancreatic duct-derived cells demonstrated a significant decrease in the *in vitro* and *in vivo* tumor cell proliferation. Furthermore, CXCR2 antagonists showed selective growth inhibition of *KRAS^(G12D)^*-bearing cells *in vitro*. Intriguingly, both genetic and pharmacological inhibition of CXCR2 signaling in *KRAS^(G12D)^*-bearing pancreatic ductal cells reduced the levels of KRAS protein, strongly implying the presence of a KRAS-CXCR2 feed-forward loop. Together, these data demonstrate the role of CXCR2 signaling in *KRAS^(G12D)^*-induced growth transformation and progression in PDAC.

## INTRODUCTION

High mortality in pancreatic cancer (PC) can be mainly attributed to its aggressive behavior and late clinical detection [1]. Pancreatic ductal adenocarcinoma (PDAC) is the most frequent (90%) histological subtype of PC and is known to arise in a step-wise manner from precursor lesions, collectively known as pancreatic intraepithelial neoplasia (PanINs) [2]. Elucidating the molecular entities that regulate the early stages, like PanINs, may facilitate the development of early diagnostic strategies and therapeutic targeting for PC.

*KRAS*, a member of *RAS* family of GTPases, is known to be mutated in 95% of the cases of PDAC. The predominant version of this earliest tumor-promoting mutation is the substitution of Glycine to Aspartic acid at codon 12 (*KRAS^(G12D)^*). Untill now, the strategies to pharmacologically block the aberrant RAS functions have turned futile in clinics [3]. Thus, to develop alternative approaches to target KRAS-induced PDAC initiation and progression, it is requisite to understand the molecular intermediaries that execute the actions of mutant *KRAS*.

In addition to the initial genetic mutations, inflammation serves as an ancillary process for the development and progression of PC [4]. The significance of inflammatory mediators such as cytokines and chemokines in cancer is well established [5, 6]. For instance, cytokines like Interleukin 4 and 13 are known to mediate important biological effects in cancer such as tumor cell proliferation, survival, adhesion and metastasis [7]. Chemokines, small molecular weight cytokines, constitute another important class of inflammatory regulators [8]. Due to the specificity of their association with cancer, cytokines and chemokines show promise to serve as diagnostic and therapeutic markers for this disease [8, 9]. Interestingly, ELR motif positive (ELR^+^) CXC chemokines are known targets of oncogenic RAS signaling [10]. ELR^+^ CXC chemokines include the ligands CXCL1-3, 5, 6, 7 and 8 and are known to bind a seven transmembrane G-protein coupled receptor (GPCR), CXCR2. However, CXCL6 and 8 are also known to bind to CXCR1, having structural homology with CXCR2 [11]. CXCR2 signaling is known to contribute to tumor progression in various cancers by promoting tumor cell growth, angiogenesis and infiltration of immunosuppressive cells in the tumor microenvironment [12, 13]. Higher expression of CXCL5 and CXCL8 has been reported in PC patient-derived tumor samples [14, 15]. In fact, CXCL5 was detected in precursor PanIN lesions of human PC tissue, suggesting its role in the early stages of the disease [16]. A recent study by Matsuo et al. addressed the role of *KRAS^(G12D)^* in the upregulation of cumulative expression of hCXCL1, 5 and 8 and identified their non-tumor-cell-autonomous role in the context of *KRAS^(G12D)^*-induced growth transformation [17]. However, some of the earlier research efforts have indicated the role of CXCR2 signaling in mediating autonomous growth of tumor cells in PC [18–20]. Expression of both CXCR2 and its ligands is detected on PC cell lines [19]. More importantly, CXCR2 ligands CXCL1 and CXCL8 have been reported to be autocrine growth factors for human PC cell lines [18, 20]. Disruption of the CXCR2 macromolecular complex was found sufficient to inhibit the proliferation of PC cells *in vitro* and *in vivo* [19]. Growth stimulatory downstream signaling of RAS protein is primarily mediated by the activation of the ERK pathway [21]. GPCRs are known to regulate cell growth by activating MAPK pathway via RAS [22]. Interestingly, CXCR2 signaling is also known to induce activation of ERK pathway [23]. More specifically, reports in gastric cancer and melanoma provide evidence for the direct role of CXCL1 (a CXCR2 ligand) in regulating the protein levels of KRAS [24, 25].

Taken together, these lines of evidence strongly support the theory that CXCR2 signaling might play an important role in KRAS-induced tumor cell-autonomous growth by directly contributing to its intracellular signaling during PDAC development and progression. Therefore, the objective of the current study was to investigate the autocrine role of CXCR2 signaling in regulating *KRAS^(G12D)^*-induced growth transformation in PDAC. Our data demonstrates that CXCR2 signaling mediates KRAS-induced PC growth and suggests that targeting CXCR2 signaling might be a feasible approach to inhibit *KRAS^(G12D)^*-induced PDAC tumor cell growth.

## RESULTS

### Enhanced expression of CXCR2 and its ligands in the cancerous lesions of Pdx1-cre;LSL-*Kras^(G12D)^* mice

**Figure 1:**
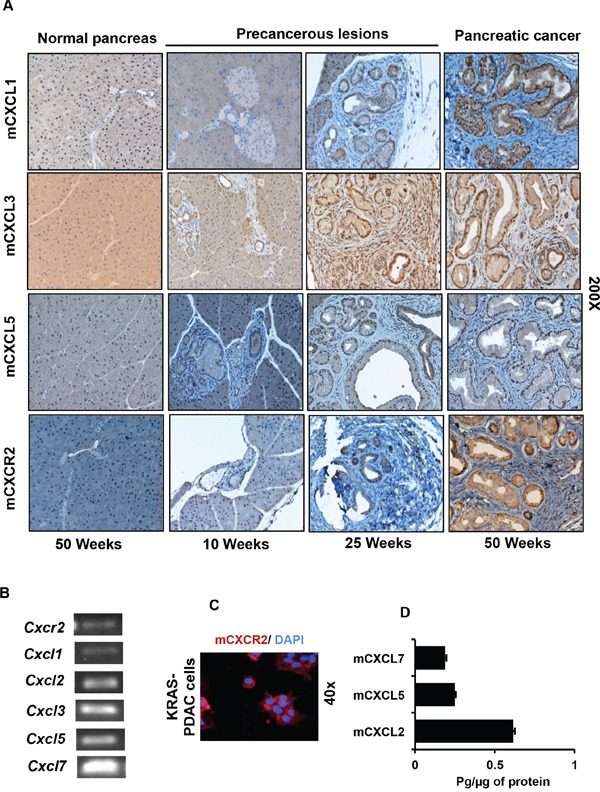
Expression of CXCR2 and its ligands progressively increases in the developing cancerous lesions of Pdx1-cre;LSL-*Kras^(G12D)^* mouse model **A.** Representative photomicrograph of immunohistochemistry performed on progression model derived from tumors of Pdx1-cre;LSL-*Kras^(G12D)^* mice at different ages (*n* = 5 mice per group), demonstrating progressively increasing expression of mCXCL1, mCXCL3, mCXCL5 and mCXCR2. The normal pancreas is negative. **B.** Expression of transcripts of *Cxcr2* and its ligands *Cxcl1, 2, 3, 5* and *7* in the KRAS-PDAC cells. **C.** Immunofluorescence for detection of mCXCR2 on KRAS-PDAC cells. **D.** Expression of mCXCL2, 5 and 7 in culture supernatants of KRAS-PDAC cells, as measured by ELISA.

As most of the reports in PC have used *in vitro* cell line model systems, the precise spatiotemporal pattern for expression of CXCR2 and its ligands in the context of introducing the *KRAS^(G12D)^* mutation *in vivo* remains unclear [17]. Therefore, we used Pdx1-cre;LSL-*Kras^(G12D)^* mouse model having a pancreas-specific expression of the *Kras^(G12D)^* mutation [26]. Pancreatic tissues derived from mice sacrificed at different time points (10, 25 and 50 weeks age) were used to generate a progression model. We observed no expression of mCXCR2 and its ligands mCXCL1, 3 and 5 in the pancreas, derived from the control Pdx1-cre mice. However, in Pdx1-cre;LSL-*Kras^(G12D)^* mice beginning at 10 weeks of age, expression of mCXCR2, mCXCL1, 3 and 5 was observed (Figure 1A). This expression was further intensified in the tumors of mice at 25 and 50 weeks age. The expression was localized in both PDAC (ductal) cells as well as the surrounding stroma. Supplementary Figure S2A to S2D provide representative photographs at both lower and higher magnification demonstrating the same results. The PDAC cell-specific expression of mCXCR2 was further confirmed by performing dual immunofluorescent staining for cytokeratin and mCXCR2 (Supplementary Figure S2E).

To establish an *in vitro* system for further experimentation, we used PDAC cells isolated from Pdx1-cre;LSL-*Kras^(G12D)^* mice as described previously [27]. We confirmed the expression of transcripts for *Cxcr2* and *Cxcl1, 2, 3, 5* and *7* in the KRAS-PDAC cells by PCR (Figure 1B). Expression of CXCR2 protein was confirmed by immunofluorescence (Figure 1C). ELISA of culture supernatants of KRAS-PDAC cells detected mCXCL5 that was also detected by IHC. Furthermore, two additional ligands mCXCL2 and 7 were also detected by ELISA (Figure 1D). Collectively, these data demonstrate that: a) expression of mCXCR2 and its ligands (mCXCL1, mCXCL3 and mCXCL5) progressively intensifies in the developing lesions of Pdx1-cre;LSL-*Kras^(G12D)^* mice; and b) ductal cells express mCXCR2 and its ligands both *in vivo* and *in vitro*.

### *KRAS^(G12D)^* mutation-bearing human pancreatic cancer cells show higher expression of CXCR2 and its ligands

**Figure 2:**
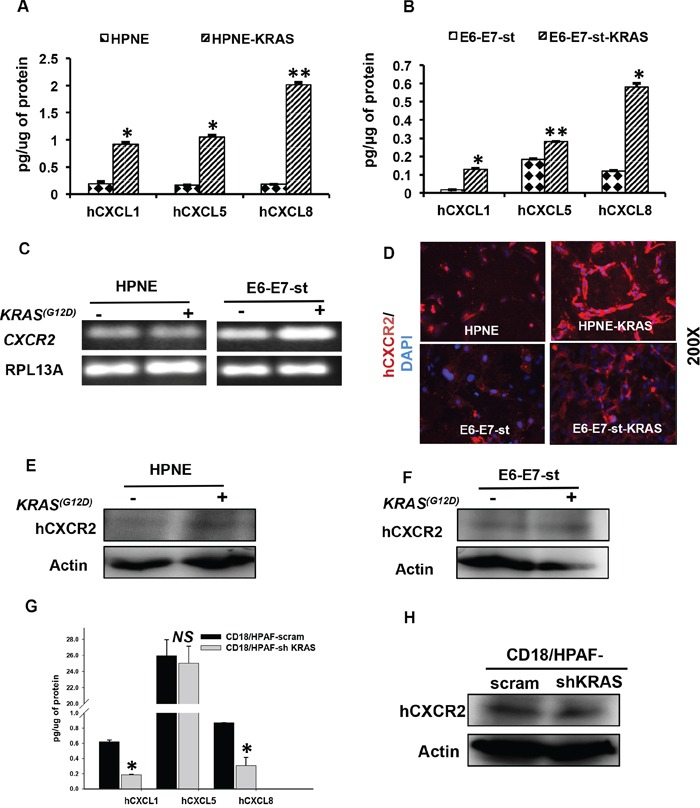
The *KRAS^(G12D)^* mutation regulates the expression of CXCR2 and its ligands in human pancreatic cancer cells Expression levels of hCXCL1, 5 and 8 in culture supernatants of **A.** HPNE and HPNE-KRAS **B.** E6-E7-st and E6-E7-st-KRAS cells, as detected by ELISA. Values are normalized to total μg of protein. **C.** PCR to detect the RNA transcript levels of *CXCR2* in HPNE, HPNE-KRAS and E6-E7-st, E6-E7-st-KRAS cells. **D.** Immunofluorescence for hCXCR2 in HPNE, HPNE-KRAS and E6-E7-st, E6-E7-st-KRAS cells. Western blots to detect the protein levels of hCXCR2 in whole cell lysates of **E.** HPNE, HPNE-KRAS and **F.** E6-E7-st, E6-E7-st-KRAS. **G.** ELISA to detect the levels of hCXCL1, 5 and 8 proteins in the culture supernatants of CD18/HPAF scram and CD18/HPAF-shKRAS cells. **H.** Western blots to detect CXCR2 protein levels in CD18/HPAF-scram and CD18/HPAF-shKRAS cells. Statistical significance determined by Student's t-test (*p ≤ 0.05, **p ≤ 0.01, ***p ≤ 0.001, *NS p* > 0.05)

We next assessed whether *KRAS^(G12D)^* alters the expression of CXCR2 and its ligands by using: I) immortalized human pancreatic ductal cells having exogenous expression of *KRAS^(G12D)^* [HPNE/-KRAS and E6-E7-st/-KRAS] or II) human PC cell line with deletion of endogenous *KRAS^(G12D)^*, CD18/HPAF-scram/-shKRAS. In culture supernatants of both HPNE/-KRAS and E6-E7-st/-KRAS cell line models, we detected significantly higher expression of hCXCL1, 5 and 8 in the *KRAS^(G12D)^*-bearing cells compared with their control counterparts (Figure 2A and 2B). The expression levels of RNA transcripts of *CXCL1, 2, 3, 5* and *8* were evaluated by qRT-PCR in both cell models. In the HPNE-KRAS cell line *CXCL5* was found to be significantly upregulated (Supplementary Figure S3A). However, the E6-E7-st-KRAS cells showed enhanced expression of all the *CXCR2* ligands (Supplementary Figure S3B). We next looked for the presence of CXCR2 expression in both cell line models. The E6-E7-st-KRAS cells demonstrated an upregulation of *CXCR2* RNA transcript in comparison to the control counterpart (Figure 2C). We further confirmed the enhanced expression of CXCR2 in the *KRAS^(G12D)^*-bearing cells compared with their control counterparts by immunofluorescence (Figure 2D) and Western blot (Figure 2E and 2F). ELR^+^ CXC chemokines are also known to interact with CXCR1. In order to, assess the impact of *KRAS^(G12D)^* mutation on altering CXCR1 expression we next evaluated the expression of *CXCR1* transcripts in both cell line models. We detected a higher expression of *CXCR1* in the E6-E7-st-KRAS cells compared with the control equivalents (Supplementary Figure S3C).

Furthermore, stable clones of CD18/HPAF knocked-down for *KRAS^(G12D)^* demonstrated inhibition in the secreted levels of hCXCL1, 5 and 8 in the culture supernatants (Figure 2G) and hCXCR2 in the total cell lysates (Figure 2H). Together, these data demonstrate that *KRAS^(G12D)^* mutation directly induces the expression of hCXCR2 and its ligands in the PDAC cells.

### Blocking CXCR2 signaling inhibits *KRAS^(G12D)^*-induced *in vitro* cell growth and migration

**Figure 3:**
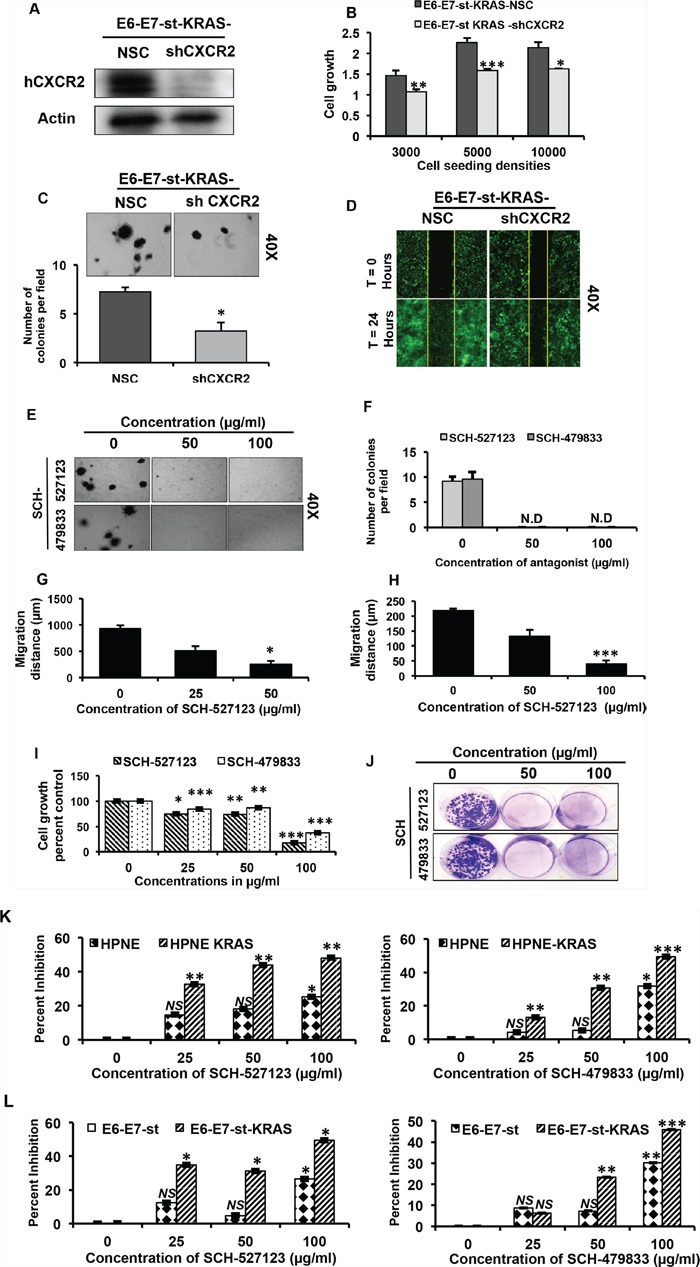
CXCR2 signaling mediates *KRAS^(G12D)^*-induced autocrine cell growth and migration **A.** Western blot of total cell lysates of E6-E7-st KRAS-NSC/-shCXCR2 cells, demonstrating deletion of hCXCR2 at the protein level in the knock-down cells. **B.** Cell viability of E6-E7-st-KRAS-NSC/-shCXCR2 cells at different seeding densities at 72 hours evaluated by MTT assay **C.** Anchorage-independent growth potential of E6-E7-st-KRAS-NSC/-shCXCR2 cells evaluated by soft agar colony formation assay. **D.** Wound healing assay to assess the migratory potential of E6-E7-st-KRAS-NSC/-shCXCR2 cells. **E.** Anchorage-independent growth potential of E6-E7-st-KRAS cells evaluated by soft agar colony formation assay treated with indicated doses of SCH-527123 or SCH-479833 for 2 weeks. **F.** Quantitation of colonies in soft agar assay. Quantitation of wound healing assay performed on **G.** HPNE-KRAS and **H.** E6-E7-st-KRAS cells treated with indicated doses of SCH-527123 or SCH-479833 in serum-free medium for 24 hours. **I.** Cell viability of KRAS-PDAC cells treated with SCH-527123 or SCH-479833 for 72 hours evaluated by MTT assay. **J.** Anchorage-dependent clonogenic potential of KRAS-PDAC cells treated with SCH-527123 or SCH-479833. **K.** and **L.** CXCR2 antagonists provide selective growth disadvantage to the mutant KRAS-bearing cells versus the wild-type KRAS-bearing cells. Percent inhibition in cell viability evaluated by MTT assay for HPNE, HPNE-KRAS (K) and E6-E7-st, E6-E7-st-KRAS (L) cells incubated with the indicated doses of SCH-527123 or SCH-479833 for 72 hours. Statistical significance determined by paired Student's t-test (*p ≤ 0.05, **p ≤ 0.01, ***p ≤ 0.001, *NS p* > 0.05).

The goal of the next set of our experiments was to evaluate whether the inhibition of CXCR2 signaling modulates *KRAS^(G12D)^*-induced autocrine cell growth. To investigate this, we generated stable CXCR2 knock-down clones of E6-E7-st-KRAS cells (Figure 3A). Knocking-down CXCR2 significantly inhibited the *in vitro* cell viability (Figure 3B) and anchorage-independent growth (Figure 3C). Furthermore, E6-E7-st-KRAS-shCXCR2 cells demonstrated markedly reduced *in vitro* cell migration potential (Figure 3D).

We next employed the pharmacological approach to inhibit CXCR2 signaling by using the antagonists SCH-527123 [11] and SCH-479833 [28]. Treatment with CXCR2 antagonists induced a dose-dependent suppression of anchorage-independent growth in the transformed E6-E7-st-KRAS cells (Figure 3E and F). We also observed an inhibitory influence of CXCR2 antagonists on the *in vitro* cell migration of the HPNE-KRAS (Figure 3G) and E6-E7-st-KRAS cells (Figure 3H). Representative photographs for wound healing assay are provided in supplementary Figure 4A. Furthermore, dose-dependent inhibition of cell viability (Figure 3I) and anchorage-dependent clonogenicity (Figure 3J) was observed in the KRAS-PDAC cells on treatment with the CXCR2 antagonists.

**Figure 4:**
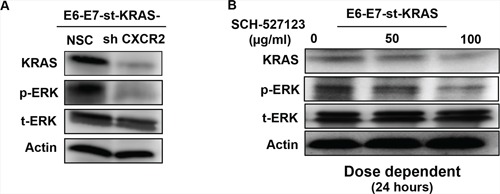
CXCR2 regulates levels of KRAS as a feed-forward loop Western blots of whole cell lysates of **A.** E6-E7-st-KRAS-NSC and E6-E7-st-KRAS-shCXCR2 cells and **B.** E6-E7-st-KRAS cells treated with indicated doses of SCH-527123 for 24 hours demonstrating the protein levels of KRAS, p-ERK and t-ERK. Actin serves as a loading control.

Since RAS is a crucial signaling pathway known to regulate the homeostatic proliferation of normal cells, it was important for us to evaluate if the CXCR2 antagonists provide a selective growth disadvantage to mutant KRAS-bearing tumor cells versus normal cells [29]. To examine this, we treated the two cell models, HPNE/-KRAS and E6-E7-st/-KRAS, with CXCR2 antagonists and evaluated percent inhibition in cell viability at a time point of 72 hours. As demonstrated in Figure 3K and 3L, at lower doses of CXCR2 antagonists there was a significant distinct difference in the growth inhibition of the *KRAS^(G12D)^*-bearing cells versus the control counter parts. Furthermore, this trend persisted at the highest dose as well. Monoclonal neutralizing antibody for CXCR2 demonstrated a similar trend (Supplementary Figure S4B). Taken together, these results demonstrate the role of CXCR2 signaling in *KRAS^(G12D)^*-induced autocrine cell growth and the specificity of CXCR2 antagonists in facilitating growth inhibition in *KRAS^(G12D)^*-bearing cells versus the control counterparts.

### Inhibiting CXCR2 signaling alters KRAS protein levels and inhibits the activation of the ERK pathway

We next evaluated how CXCR2 signaling influenced the levels of KRAS protein and activation of its downstream effectors. Our results demonstrate reduced protein levels of KRAS in E6-E7-st-KRAS-shCXCR2 versus E6-E7-st-KRAS-NSC cells as evaluated by Western blotting. Furthermore, E6-E7-st-KRAS-shCXCR2 cells showed decreased levels of p-ERK due to reduced activation of the downstream ERK pathway (Figure 4A). Treatment of E6-E7-st-KRAS cells with increasing doses of SCH-527123 (for 24 hours) demonstrated a similar trend for the expression of KRAS and p-ERK protein in a dose-dependent manner (Figure 4B).

### CXCR2 knock-down in KRAS^(G12D)^-bearing pancreatic cancer cells affects tumor growth in subcutaneous and orthotopic implants

**Figure 5:**
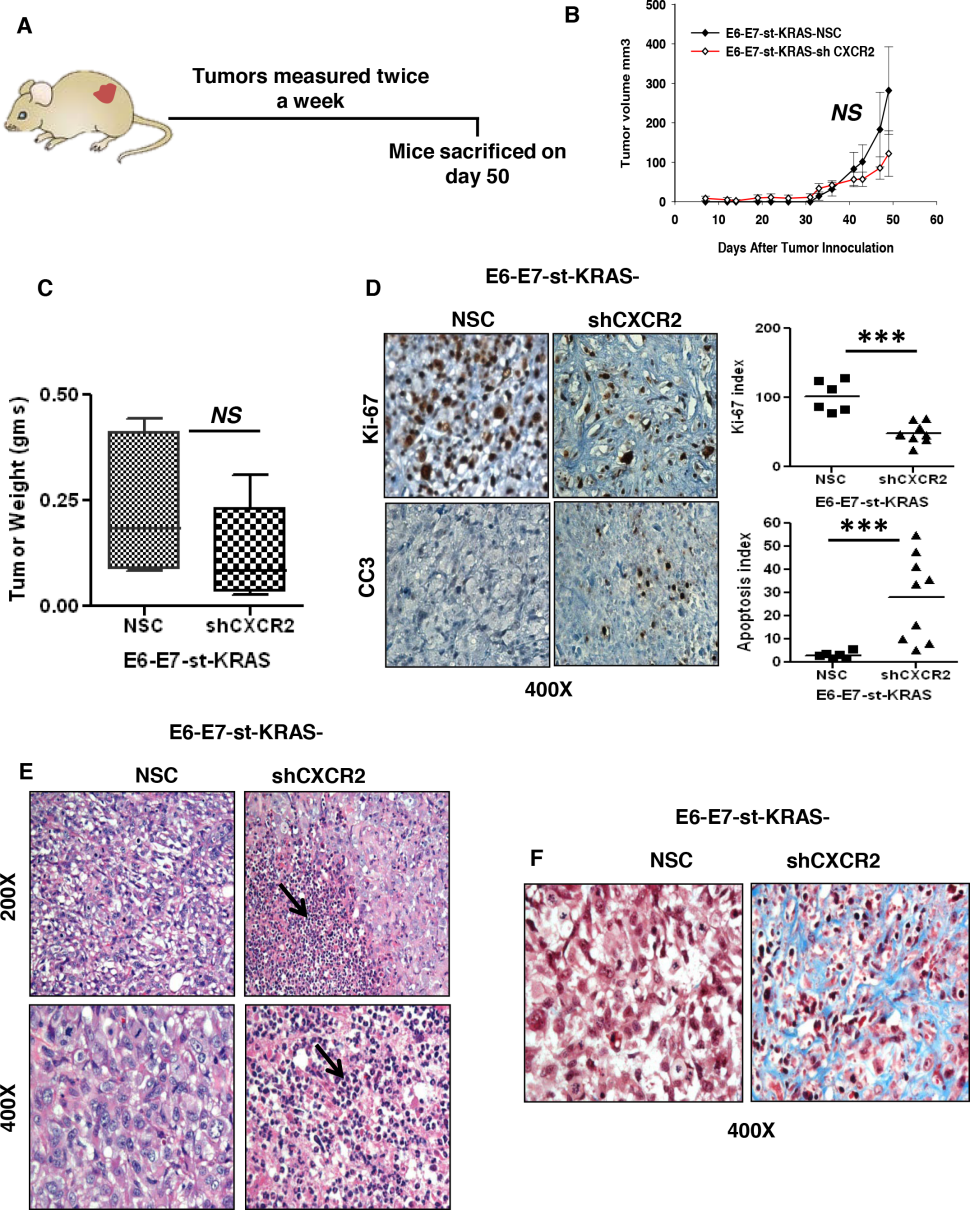
Knock-down of CXCR2 results in inhibited growth of E6-E7-st-KRAS cells in the subcutaneous implants **A.** E6-E7-st-KRAS-NSC and E6-E7-st-KRAS-shCXCR2 cells were engrafted subcutaneously in the flanks of nude mice and tumors were measured twice weekly. **B.** Tumor growth represented by the change in tumor volume (mm^3^) of subcutaneous tumors at indicated time points after inoculation. **C.** The mean weight of tumors derived from mice bearing either E6-E7-st-KRAS-NSC or E6-E7-st-KRAS-shCXCR2 cells. **D.** Representative images of immunohistochemical (IHC) staining for Ki-67 and cleaved caspase3 (CC3) in tumors of mice bearing E6-E7-st-KRAS-NSC or E6-E7-st-KRAS-shCXCR2 cells. IHCs were quantified as the average of positive cells in five independent fields per tumor at 400X. **E.** H&E staining showing infiltration of leukocytes in tumors derived from mice bearing either E6-E7-st-KRAS-NSC or E6-E7-st-KRAS-shCXCR2 cells. **F.** Masson's trichrome stain demonstrating collagen deposition. Statistical significance determined by paired Student's t-test (for tumor volume) and non-parametric Mann-Whitney *U* test. (*p ≤ 0.05, **p ≤ 0.01, ***p ≤ 0.001, *NS p* > 0.05).

For the next set of our experiments we performed subcutaneous injections of E6-E7-st-KRAS-NSC and E6-E7-st-KRAS-shCXCR2 cells in to the flanks of nude mice and measured the tumors twice a week for 50 days (Figure 5A). The E6-E7-st-KRAS-shCXCR2 cells demonstrated a non-significant (*NS*) reduction in tumor growth compared to the E6-E7-st-KRAS-NSC cells (Figure 5B). We found a reduction in the mean weight of tumors from E6-E7-st-KRAS-shCXCR2 cells compared with tumors from the control E6-E7-st-KRAS-NSC cells (*NS*) (Figure 5C). Furthermore, a decreased proliferation index and an enhanced apoptotic index, was observed by quantification of the IHCs for Ki-67 and CC3, respectively in the tumors from E6-E7-st-KRAS-shCXCR2 cells (Figure 5D). Interestingly, we observed increased infiltration of polymorphonuclear leukocytes (Figure 5E) and higher fibrosis (Figure 5F) in the E6-E7-st-KRAS-shCXCR2 tumors compared with the tumors from control cells.

**Figure 6:**
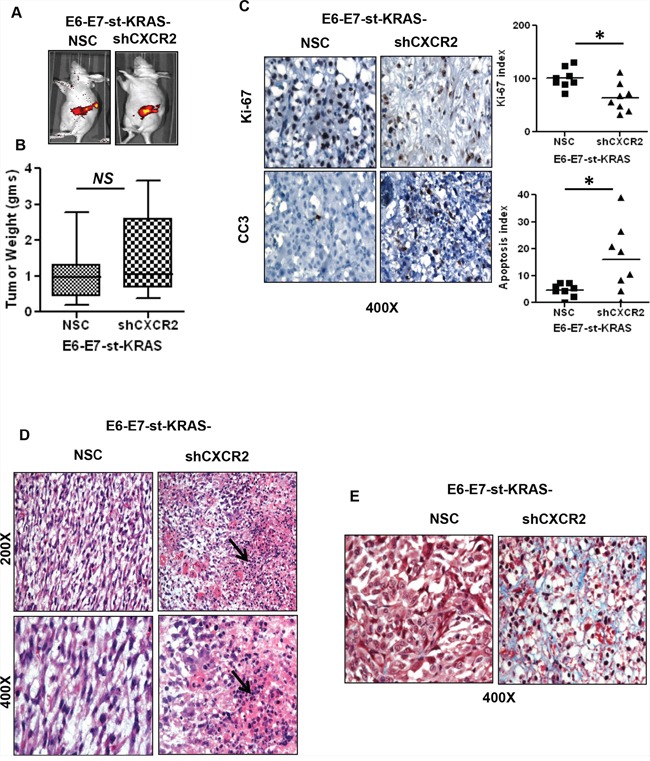
Orthotopic implants of CXCR2 knock-down cells demonstrate inhibited proliferation of tumor cells and increased fibrosis **A.** *In vivo* GFP images of E6-E7-st-KRAS-NSC or E6-E7-st-KRAS-shCXCR2 cells implanted orthotopically in the pancreas of nude mice. **B.** The mean weight of tumors derived from E6-E7-st-KRAS-NSC or E6-E7-st-KRAS-shCXCR2 cells. **C.** Representative images of immunohistochemistry (IHC) and quantified stain score for Ki-67 and cleaved caspase 3 (CC3). IHCs were quantified as the average of positive cells in five independent fields per tumor at 400X. **D.** H&E staining demonstrating infiltration of leukocytes in tumors derived from mice bearing either E6-E7-st-KRAS-NSC or E6-E7-st-KRAS-shCXCR2 cells. **E.** Masson's trichrome stain showing collagen deposition. Statistical significance determined by paired Student's t-test (*p ≤ 0.05, **p ≤ 0.01, ***p ≤ 0.001, *NS p* > 0.05).

To evaluate the effect of organ-specific responses, we implanted E6-E7-st/KRAS-NSC and E6-E7-st/KRAS-shCXCR2 cells in to the pancreas of nude mice (Figure 6A). The mean tumor weight was higher (NS) in the orthotopic implants of E6-E7-st/KRAS-shCXCR2 cells (Figure 6B). However, the tumors from E6-E7-st-KRAS-shCXCR2 cells demonstrated inhibited proliferation and increased apoptotic index compared with the tumors from the E6-E7-st/KRAS-NSC cells (Figure 6C). Increased infiltration of polymorphonuclear leukocytes was observed in the E6-E7-st-KRAS-shCXCR2 tumors compared with the control cells (Figure 6D). Also, like the subcutaneous implants the E6-E7-st-KRAS-shCXCR2 tumors demonstrated higher fibrosis as detected by Masson's trichrome staining (Figure 6E).

**Figure 7:**
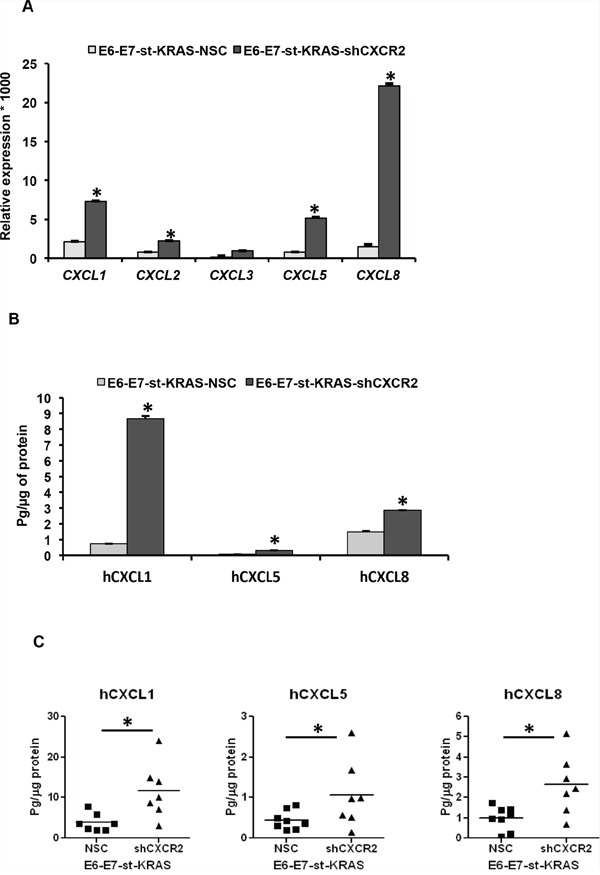
Knock-down of CXCR2 in E6-E7-st-KRAS cells upregulates the expression of its ligands **A.** Expression levels of transcripts of *CXCL1, 2, 3, 5* and *8*, normalized to *RPL13A* in E6-E7-st-KRAS-NSC and E6-E7-st-KRAS-shCXCR2 cells. **B.** Protein levels of hCXCL1, hCXCL5 and hCXCL8 in culture supernatants of E6-E7-st-KRAS-NSC and E6-E7-st-KRAS-shCXCR2 cells (24 hours). **C.** Protein isolated from tumors derived by implanting E6-E7-st-KRAS-NSC and E6-E7-st-KRAS-shCXCR2 cells orthotopically in the pancreas of nude mice were monitored for CXCR2 ligands. Statistical significance determined by paired Student's t-test and Mann-Whitney *U*-test (*p ≤ 0.05, **p ≤ 0.01, ***p ≤ 0.001, *NS p* > 0.05).

In order to find a potential explanation for the enhanced stromal responses in tumors dervied from E6-E7-st-KRAS-shCXCR2 cells, we analyzed the expression levels of transcripts of CXCR2 ligands in the E6-E7-st-KRAS-NSC and shCXCR2 cells. We observed that the E6-E7-st-KRAS-shCXCR2 cells demonstrated significantly enhanced expression of *CXCL1, 2, 3, 5* and *8* in comparison to the control cells (Figure 7A). Furthermore, ELISA performed on the culture supernatants of E6-E7-st-KRAS-NSC/-shCXCR2 cells, and also the tumors derived from the orthotopic implants of these cells showed a significantly enhanced expression of the protein levels of hCXCL1, hCXCL5 and 8 (Figure 7B and 7C). Together, these results suggest that CXCR2 knock-down resulted in the upregulation of its ligands, which causes enhanced infiltration of immune cells and increased fibrotic response inside the tumors derived from E6-E7-st-KRAS-shCXCR2 cells.

## DISCUSSION

In the present study, we aimed to investigate the role of CXCR2 signaling in mediating *KRAS^(G12D)^*-induced autocrine growth transformation of PDAC cells. Our results lead us to three novel findings: i) CXCR2 signaling is upregulated during the primitive stages of *KRAS^(G12D)^*-induced PDAC development, ii) upregulation of CXCR2 signaling by *KRAS^(G12D)^* enhances autonomous proliferation of tumor cell in PDAC, and iii) *KRAS^(G12D)^*-induced CXCR2-CXCL axis in tumor cells upregulates the expression of KRAS protein maintaining a feed-forward loop in PDAC cells.

The contribution of inflammation in carcinogenesis is well appreciated [30]. Inflammatory mediators are known to play crucial roles in tumor progression by regulating not just the immune infiltrations in tumors but also by providing a proliferation-advantage to the tumor cells [30]. Upregulation of cytokines and chemokines is one of the consequences of RAS signaling [10]. While recent reports in PDAC have identified the KRAS-CXC chemokine axis, it remains unclear if this signaling can serve as an early biomarker for PDAC progression as KRAS mutations are required for both initiation and maintenance of PDAC [31]. The expression of mCXCR2 and mCXCL1 has been previously reported *in vivo* in tumors of Ptf1a^cre/+^;LSL-*Kras^(G12D)^* mice [32]. However, to further elaborate these findings, we here identify the kinetics of their expression utilizing Pdx1-cre;LSL-*Kras^(G12D)^* mice as a model system. Our data demonstrates the expression of mCXCR2 and its ligands mCXCL1, 3 and 5 in malignant ductal cells and stroma of the precursor lesions of Pdx1-cre;LSL-*Kras^(G12D)^* mice (PanIN-1 stage) and their further enhancement as these precursor lesions advanced to PDAC.

A recent study by Matsuo et al. reported the role of *KRAS^(G12D)^* mutation in upregulating the cumulative expression of hCXCL1, 5 and 8 in the E6-E7-st-KRAS cell line model [17]. In another report, knock-down of *KRAS^(G12D)^* in a tumor derived cell line SW1990 down-regulated the transcripts of CXCR2 ligands [33]. However, these reports have utilized cell lines having additional genetic modifications apart from the *KRAS^(G12D)^* mutation. To provide experimental evidence to link *KRAS^(G12D)^* mutation with CXCR2 signaling, we generated the human pancreatic duct-derived HPNE/-KRAS cell model having *KRAS^(G12D)^* as the only genetic alteration, representing the PanIN1 stage. Our results are consistent with previous findings, and we advance the current knowledge by providing the first evidence for the role of *KRAS^(G12D)^* mutation in upregulating the expression of not just CXCLs but also CXCR2. Taken togeather, based on the results derived from HPNE/-KRAS cell line and Pdx1-cre;LSL-*Kras^(G12D)^* mouse model we conclude that the CXCR2 signaling axis is directly linked with *KRAS^(G12D)^* mutation and thus may contribute to the PDAC development during initial stages.

Previous reports implicating *RAS* mutations for inducing the expression of CXCLs have concluded that these upregulated ligands fail to provoke any autonomous growth promoting effects on the cancer cells and mediate paracrine effects by interacting with the tumor microenvironment. Using the Hela cell line, Sparmann and Sagi demonstrated that *HRAS^V12^*-induced hCXCL8-mediated tumorigenesis by enhancing angiogenesis [34]. In ovarian cancer *HRAS^V12^*-induced upregulation of hCXCL1 was found to promote tumor growth through the induction of senescence in the stromal fibroblasts [35]. Furthermore, a report in lung cancer demonstrated that *KRAS^(G12D)^*-induced CXCLs mediated tumorigenesis by recruiting inflammatory and endothelial cells [36]. More relevant to the current study, recent reports in PC have implicated *KRAS^(G12D)^*-induced CXCLs as mediators of angiogenesis [17] or fibrosis [32] and reported a lack of autocrine growth promoting effects on PC cells. Succinctly, the two fundamental reasons for the absence of CXCLs mediated autocrine effects in all these studies were: a) lack of the receptor CXCR2 on these cells; and b) dysfunctionality of the receptor. However, numerous data support the theory of CXCR2-mediated tumor cell autochthonous growth in PC. Takamori et al. identified the expression of CXCR2 and its ligands CXCL1 and 8 on Capan-1 cells [18]. They found that treatment with anti-CXCL8 or anti-CXCL1 antibody inhibited the growth of Capan-1 cells. In line with these observations, Kamohara et al. also demonstrated that neutralizing antibody for CXCL8 (1-100 μg/ml) was sufficient to significantly suppress the autocrine growth of PC cell lines, including SUIT-2 and Capan-1 [37]. Furthermore, while identifying the presence of CXCR2 macromolecular signaling complex in PDAC cells, a recent study provided additional functional evidence for the role of CXCR2 signaling in mediating *in vitro* and *in vivo* tumor cell growth. Their data revealed that treatment of PDAC cell lines HPAC and Colo357, with CXCR2 agonists CXCL1, 5 and 8 enhanced their *in vitro* cell proliferation. Similarly, disruption of the CXCR2 macromolecular complex by using an exogenous CXCR2 C-tail sequence in HPAC cells significantly attenuated the *in vitro* and *in vivo* proliferation [19].

As we established the presence of CXCR2 receptor as well as its ligands in *KRAS^(G12D)^*-bearing PDAC cell models we hypothesized the possible existence of a self-sufficient CXCR2 signaling loop on PDAC cells, which may act as a mediator of *KRAS^(G12D)^*-induced autocrine growth transformation. One of the main findings of the current study was that inhibiting CXCR2 genetically or pharmacologically decreased *KRAS^(G12D)^*-induced tumor cell growth. Knocking-down CXCR2 in E6-E7-st-KRAS demonstrated a significant growth inhibition *in vitro* and *in vivo.* Tumors obtained from subcutaneous and orthotopic implants showed reduced cell proliferation and enhanced apoptosis in the shCXCR2 tumors versus the tumors of control cells. However, the enhanced weight of the tumors derived from E6-E7-st-KRAS-shCXCR2 cells compared with the tumors of control cells in orthotopic implants can be attributed to the fact that the CXCR2 knock-down increased the expression of allied ligands in these cells resulting in paracrine effects like infiltration of immune cells and fibrosis.

Taken together, these results indicate that *KRAS^(G12D)^*-induced expression of CXCR2 and its ligands mediate autocrine growth transformation in PDAC. Two facts can mainly explain the inconsistency of these findings with previous reports linking CXCR2 signaling with *KRAS^(G12D)^* mutation. Firstly, they have used HPDE cell line versus the HPNE cell line used in our study. Secondly, unlike the *KRAS^(G12D)^* mutation in our report they have studied the effects of *K-Ras4B^G12V^* mutation [17]. Of note, it has been reported that not all mutant KRAS proteins cause the downstream signaling in a similar way, which may lead to different functional patterns [38]. In a recent study Ijichi et al. reported that CXCR2 inhibition in mPanIN cell lines isolated from Ptf1a^cre/+^;LSL-*Kras^(G12D)^* mice demonstrated no inhibition of cell growth [32]. The contrariety of their findings with our results can be explained by the fact that we have used cell lines isolated from Pdx1-cre;LSL-*Kras^(G12D)^* mice, which employs a Pdx1 promoter for inducing the expression of *Kras^(G12D)^* versus the Ptf1a promoter utilized by them.

We also demonstrate that pharmacological inhibition of CXCR2 by two antagonists SCH-527123 and SCH-479833 [11] engenders selective growth inhibition and toxicity on the *KRAS^(G12D)^*-bearing cells versus the normal RAS-bearing control cells. We have previously reported anti-tumor and anti-metastatic effects of CXCR2-antagonists in melanoma [28] and colon cancer [39] respectively. Additionally, in a recent study by *Y Ning* et al. SCH-527123 was shown to demonstrate *in vitro* and *in vivo* antitumor effects either alone or in combination with Oxaliplatin in colon cancer [40]. In a proof-of-principle study on ozone-challenged healthy human subjects, SCH-527123 was found to inhibit pulmonary neutrophilia. Importantly, with only a few mild adverse effects, the oral administration of SCH-527123 was well tolerated by human subjects [41].

Activation of ERK pathway has been implicated in mediating RAS-induced autocrine and paracrine cell growth [21]. To understand how CXCR2 inhibition causes a reduction in the *KRAS^(G12D)^*-induced growth potential of PDAC cells we evaluated the activation of the ERK pathway and total levels of KRAS. Our results demonstrated a marked reduction in levels of p-ERK and KRAS after inhibiting the CXCR2 signaling in the E6-E7-st-KRAS cells either genetically or pharmacologically. These findings are in agreement with previous reports that have identified the role of CXCR2 signaling in regulating the levels of KRAS protein [24, 25] and activation of the ERK pathway [23]. In a study performed to evaluate gastric cancer metastasis Cheng et al. reported that the ectopic expression of CXCL1 or knock-down of endogenous CXCL1, in a gastric cancer cell line AAZ521 respectively upregulates or downregulates the expression of KRAS protein in the total cell lysates [25]. Likewise, a study in melanoma showed that CXCL1-mediated melanocyte transformation involves the induction of RAS expression. CXCL1-mediated transformation of clones of immortalized murine melanocytes resulted in elevated levels of KRAS protein and also enhanced RAS activation [24].

**Figure 8:**
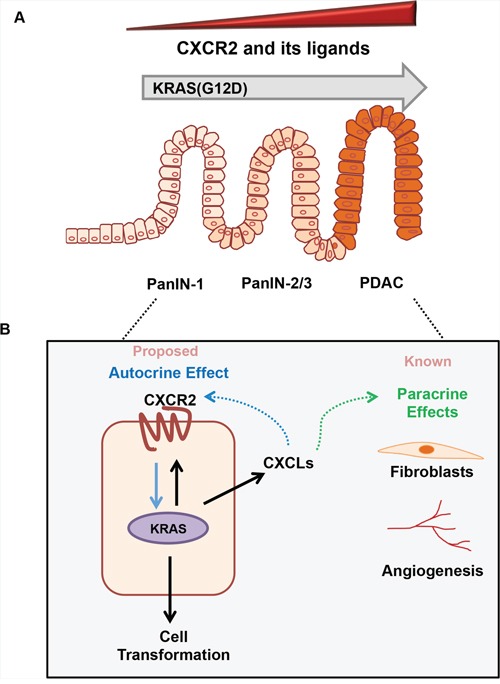
The role of CXCR2 signaling in *KRAS^(G12D)^*-induced development of pancreatic cancer Based on data in the current study, and previously published reports we can summarize the role of CXCR2 signaling in KRAS-induced initiation and progression of pancreatic cancer as follows: **A.** The point mutation in KRAS gene induces CXCR2 and its ligands. Their expression is detected early and further enhances as the precursor lesions pancreatic intraepithelial neoplasia (PanINs) advance to pancreatic ductal adenocarcinoma (PDAC). **B.** The enhanced KRAS activity inside the ductal cell induces the expression of both CXCR2 and CXCLs. Previous reports in pancreatic cancer identify how CXCR2 ligands indirectly alter the tumor progression by affecting endothelial cells and fibroblasts in the tumor microenvironment. In the current study, we propose a novel cell-autonomous model where these upregulated CXCLs bind to CXCR2 receptor on the surface of the ductal cells. This CXCR2-CXCL autocrine loop in turn reinforces the expression of KRAS protein and enhances the growth of tumor cells.

In summary, the current study highlights a novel role of CXCR2 signaling in mediating *KRAS^(G12D)^* mutation-induced autocrine growth transformation of tumor cells by directly modulating the levels of KRAS protein and its downstream signaling (Figure 8). We anticipate that further research based on the data provided in the current study may enable the development of clinically effective treatments for KRAS-induced PDAC in future.

## MATERIALS AND METHODS

### Pdx1-cre;LSL-*Kras^(G12D)^* mice and KRAS-PDAC cells

Pdx1-cre;LSL-*Kras^(G12D)^* mice described previously in [26] were used for the study. Mice were sacrificed at time points of 10, 25 and 50 weeks age. Tissue specimens were collected, paraffin embedded and sectioned. Generation and characterization of the UN-KC-6141 cell line (referred to as KRAS-PDAC cells in this study) have been previously described [27]. The KRAS-PDAC cells were maintained in Dulbecco's Modified Eagle Medium (DMEM) (HyClone^®^, Thermo Scientific, UT) supplemented with fetal bovine serum (Atlanta Biologicals, GA), L-Glutamine (MediaTech, VA), twofold vitamin solution (MediaTech) and Gentamycin (Gibco, Life Technologies, NY).

### Cell lines and cell culture

We used a model of immortalized human pancreatic duct-derived cell lines, with or without exogenous expression of *KRAS^(G12D)^*. The model consisted of four cell lines hTERT-HPNE (HPNE), hTERT-HPNE-KRAS^(G12D)^ (HPNE-KRAS) [both cell lines together referred to as HPNE/-KRAS], hTERT-HPNE-E6/E7/st (E6-E7-st) and hTERT-HPNE-E6/E7/st-KRAS^(G12D)^ (E6-E7-st-KRAS) [both cell lines together referred to as E6-E7-st/-KRAS]. Generation and maintenance of hTERT-HPNE, E6-E7-st and E6-E7-st-KRAS cells have been previously described [42].

To reconstitute this first stage of the disease, hTERT-HPNE cells were modified to express oncogenic *KRAS^(G12D)^* (Supplementary Figure S1). Additionally, CD18/HPAF cells with knock-down of the endogenous *KRAS^(G12D)^* allele were used (CD18/HPAF-scram (control), CD18/HPAF-shKRAS). Generation and growth conditions of these cells are described earlier [43].

### Generation of CXCR2 knock-down cells

Six individual clones of human GIPZ lentiviral shRNAmir anti-CXCR2 were obtained from Thermo Scientific Open Biosystems (Grand Island, NY). A scrambled shRNA was used as a non-silencing control (NSC). Lentiviral particles were generated by us and cells were infected according to the manufacturer's protocol. Stable knock-down of CXCR2 was achieved in E6-E7-st-KRAS cells by pooling together the six different anti-CXCR2 shRNA individual clones.

### Reagents and antibodies

The two CXCR2 antagonists SCH-527123 and SCH-479833 were obtained from Schering-Plough Research Institute and were dissolved in 20% hydroxypropyl-β-cyclodextrin (HPβCD) from Acros Chemical (St. Louis, MO). All the antibodies used in the present study are listed in Supplementary Table 1.

### Enzyme-linked immunosorbent assay (ELISA)

Equal numbers (1 × 10^6^) of cells CD18/HPAF-scram (control), CD18/HPAF-shKRAS, HPNE, HPNE-KRAS, E6-E7-st, E6-E7-st-KRAS, E6-E7-st-KRAS-NSC, E6-E7-st KRAS-shCXCR2 and KRAS-PDAC cells were plated in 60 mm dishes in complete medium. After attachment of cells to the plate, the medium was changed to serum-free DMEM. Supernatants of cultured cells were collected at 24 hours or 72 hours. Protein was isolated from tumors by homogenizing in a bullet blender using Mammalian Protein Extraction Reagent (Pierce, Rockford, IL) as a lysis buffer. ELISA assays for hCXCL8 and hCXCL1 were performed as described previously [39]. hCXCL5, mCXCL2, mCXCL5 and mCXCL7 ELISAs were performed using a duoset kit (R&D Systems, Minneapolis, MN) according to the manufacturer's protocol. All the experiments were performed in duplicates.

### Immunohistochemistry

4 μm thick, formalin-fixed, paraffin-embedded tissue sections were deparaffinized. Antigen retrieval was performed using sodium citrate buffer (pH = 6.0) and microwaving for 10 minutes. Endogenous peroxidase was blocked by incubating with 3% hydrogen peroxide in methanol for 30 minutes. After blocking non-specific binding by incubating with serum, slides were probed with appropriate primary antibodies (listed in Supplementary Table 1) overnight at 4°C. Slides were washed, and appropriate secondary antibodies were added. Immunoreactivity was detected using the ABC Elite Kit and 3, 3 diaminobenzidine substrate kit (Vector Laboratories, Burlingame, CA) as per the manufacturer protocols. A reddish brown precipitate indicated positive staining. Nuclei were counterstained with hematoxylin. To evaluate cell proliferation and apoptosis the number of Ki-67 and cleaved caspase 3 (CC3) positive cells respectively were counted in five independent areas at 400 X.

### Cell viability assay

Cells were seeded at indicated densities in 96-well plates and were allowed to adhere. Cells were washed with HBSS and were incubated with medium alone or medium containing specified concentrations of the CXCR2 antagonists for 72 hours. Cell viability was determined by MTT assay (3-(4, 5 dimethylthiazol-2-yl)-2, 5-dipehnyltetrazolium bromide, tetrazole) as previously described [44].

Percent inhibition in cell growth was calculated by the formula: [100 - (A/B) × 100], where ‘A’ and ‘B’ are the absorbances of the treated and untreated cells, respectively. Percentage of cell growth was calculated by the formula: [(A/B) × 100], where ‘A’ and ‘B’ are the absorbances of treatment and control group respectively.

### Anchorage-dependent and -independent growth assay

To evaluate anchorage-dependent (clonogenic) potential KRAS-PDAC cells were plated at a density of 2500 cells/well in a 6-well plate and treated with different concentrations of CXCR2 antagonists in 10% DMEM. Clonogenicity was evaluated after 10 days by fixing cells in methanol and staining with crystal violet.

Anchorage-independent growth (colony formation) was assessed by plating 3000 cells per well in 0.3% agarose with a 0.6% agarose underlay in a 6-well plate. CXCR2 antagonists were added at indicated concentrations to both 0.3% agarose layer and the medium covering the 3% agarose layer. Cells were incubated for 2 weeks at 37°C in a 5% CO_2_ incubator. Media was changed once every week. Colonies were fixed in a solution of acetone with methanol and stained with 0.5% crystal violet and counted under an inverted microscope at 40X magnification.

### Cell migration assay

A wound healing assay was conducted to assess the migratory potential of the cells. Cells were plated in 60 mm dishes. After the cells had reached 90-95% confluence, a wound was generated using 1 ml pipette tip. Cells were washed with HBSS and incubated with either serum-free medium or with serum-free medium containing indicated concentrations of CXCR2 antagonists for 24 hours. Cells were photographed under an inverted microscope at 40X magnification at time T = 0 hours and T = 24 hours. The width of the wound was measured using NIH Image J software. Distance migrated was calculated by the formula: Initial wound width (T = 0 hours) - Final wound width (T = 24 hours).

### Tumor formation assay

6- to 8-week-old female nude mice were obtained from Charles River Laboratories (Wilmington, MA). Mice were maintained under specific pathogen-free conditions. All procedures performed were in agreement with institutional guidelines and approved by the University of Nebraska Medical Center Institutional Animal Care and Use Committee. E6-E7-st-KRAS-NSC and E6-E7-st-KRAS-shCXCR2 cells (1 × 10^6^ in 50 μl HBSS) were injected into the pancreas (orthotopic) or flanks (subcutaneous) of nude mice. For subcutaneous implants, the tumors were measured twice a week for 50 days with a caliper. The tumor volume was calculated using the formula: volume= (length × width^2^)/2. Subcutaneous tumors (50 days post inoculation) and orthotopic tumors (8 weeks post inoculation) were resected, fixed in 10% formalin and paraffin embedded.

### Statistical analysis

Statistical analysis was conducted using GraphPad Prism software. Error bars represent standard error of mean. The significance was determined by Student's t-test or nonparametric Mann-Whitney *U*-test. For all statistical tests, a *p*-value of ≤ 0.05 was considered significant.

### Study approval

All animal protocols were approved by the University of Nebraska Medical Center Institutional Animal Care and Use Committee (IACUC).

## SUPPLEMENTAL MATERIALS AND METHODS, FIGURES AND TABLES


