# Supplementary material for: CXCR2 signaling regulates KRAS(G12D)-induced autocrine growth of pancreatic cancer
Source: Oncotarget. 2016 Jan 13;7(6):7280–96. doi: 10.18632/oncotarget.6906 (PMC4872785; doi:10.18632/oncotarget.6906)
Supplement: Supplementary file 1 [file oncotarget-07-7280-s001.pdf]

SUPPLEMENTAL MATERIALS AND METHODS, FIGURES AND TABLES

Generation of HPNE-KRAS cells

HPNE cells were transduced with retroviral vector pLXSH carrying oncogenic *KRAS*$^{(G12D)}$, after which point cells were selected for viral integration (hygromycin 200 ug/ml). After 2 weeks of growth, cells were probed for the presence of the exogenous RAS mutant. Western blot analyses performed with an antibody (pan RAS-12 Ab-1 Cat: PC10L-100UG, EMD Millipore, MA) specific for the G12D mutation of RAS detected KRAS$^{(G12D)}$ in the transduced cells, but not in control cells transduced with an empty vector (Figure S1A). To more precisely measure the ratio between the expression of the endogenous and exogenous forms of KRAS, RT-PCR was employed. PCR primers [1] capable of amplifying both forms of the message were used to produce a PCR fragment, which was then cleaved with BccI to distinguish the wild type and mutant sequences (Figure S1B). These results indicated that we had successfully generated HPNE cells expressing *KRAS*$^{(G12D)}$, which we could use as a reconstituted model of PanIN-1 lesions.

RNA isolation and PCR analysis

Total RNA was isolated from cells using the standard Trizol (Invitrogen, Carlsbad, CA) protocol. Reverse Transcription was performed with 5 µg RNA using oligo (dT) (Fermentas, Hanover, MD) and Superscript® II RT (Invitrogen). Regular and quantitative real-time PCR reactions with primer sets (Table S2) were performed using Fast Start Taq DNA polymerase or FastStart SYBR Green Master Mix (Roche; Indianapolis IN) respectively using the MyIQ™ iCycler (Bio-Rad; Hercules, CA). For regular PCR, amplified cDNA was resolved on ethidium bromide containing agarose gels. For real-time PCR mean C_t values of the target genes were normalized to mean C_t values of the endogenous control, ribosomal protein large 13 A (RPL13A); [$-\Delta C_t = C_t$ (RPL13A) – C_t (target gene)]. The ratio of mRNA expression of target genes versus RPL13A was defined as $2^{(-\Delta Ct)}$. Melting curve analysis was performed to check the specificity of the amplified product.

Immunofluorescence

Cells were cultured on 8-well chamber slides and were allowed to adhere overnight. The following day, cells were fixed using 4% paraformaldehyde, blocked with antibody diluent (BD Biosciences, San Jose, CA), and probed with an anti-CXCR2 antibody (Table S1) (4 °C overnight). The next day, slides were incubated with a Cy3-conjugated anti-mouse antibody. Nuclei were counterstained with DAPI (4, 6 diamidino-2-phenylindole). Finally, slides were mounted with Vectashield® mounting medium (Vector Laboratories, Burlingame, CA) and observed under a fluorescent microscope.

Western blot analysis

Total protein was isolated by lysing cells in RIPA buffer. The protein concentrations were determined using BCA kit (Pierce™ BCA Protein Assay Kit (Thermo Scientific, Rockford, IL). Protein samples (40 µg or 25 µg) were electrophoresed on sodium dodecyl sulfate-polyacrylamide gels (10% or 15%) and transferred to Immobilon-p Transfer membrane (Millipore, Billerica, MA). Membranes were blocked with 3% BSA in PBS for 1 hour at room temperature. Membranes were probed with specific primary antibodies (Table S1) overnight at 4 °C. Membranes were washed with TTBS buffer, thrice and probed with respective secondary antibodies. Following washing with TTBS buffer membranes were visualized using SuperSignal® West Femto Kit (Thermo Scientific).

REFERENCE

1. Campbell PM, Groehler AL, Lee KM, Ouellette MM, Khazak V and Der CJ. K-Ras Promotes Growth Transformation and Invasion of Immortalized Human Pancreatic Cells by Raf and Phosphatidylinositol 3-Kinase Signaling. Cancer research. 2007; 67:2098–2106.

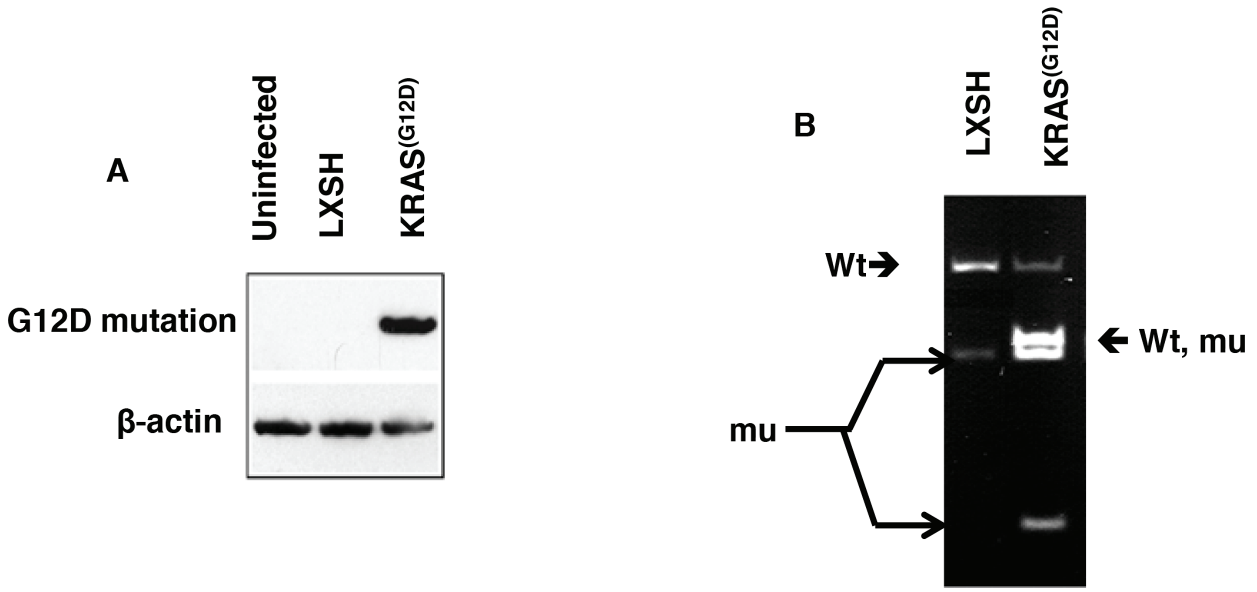

Supplementary Figure S1: Generation of HPNE cells expressing *KRAS$^{(G12D)}$*. hTERT-HPNE cells were transduced with retroviral vector pLXSH carrying no insert or carrying KRAS^{G12D}. Following hygromycin selection, cells were analyzed for the expression of RAS. **A.** Western blot for the detection of KRAS^{G12D} mutant in uninfected cells and cells infected with the KRAS mutant (*KRAS^{G12D}*) or the empty vector (LXSH). **B.** RT-PCR analysis for evaluating the relative level of wild-type and mutant KRAS expression. RNA isolated from the samples was RT-PCR amplified with primers able to recognize both forms of KRAS, after which point the PCR fragments were digested with BccI. wt: fragment derived from the wild-type sequences. mu: sequence derived from the mutant sequences.

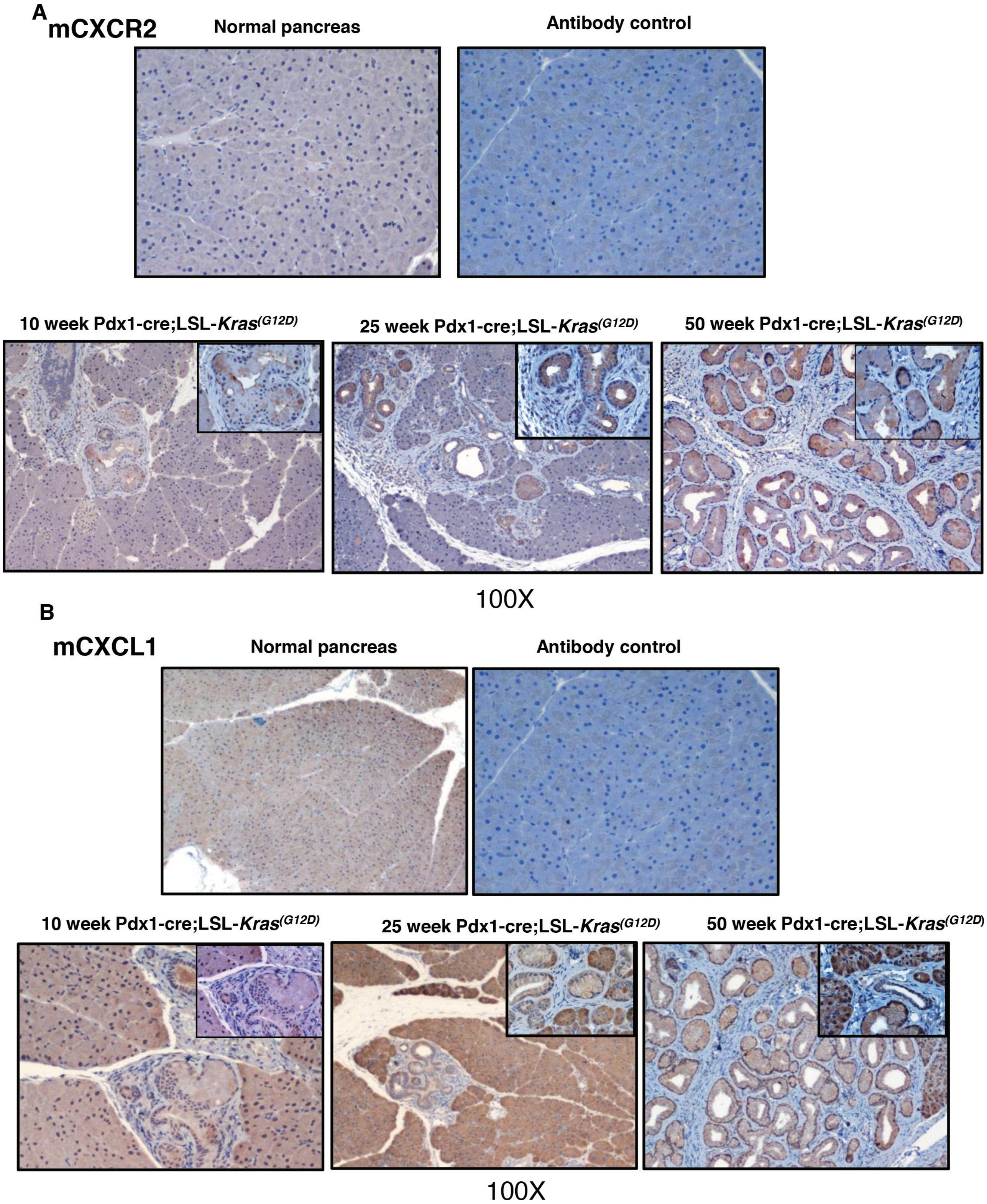

Supplementary Figure S2: Lesions from Pdx1-cre;LSL-*Kras*$^{(G12D)}$ mice showing a progressive increase of CXCR2 signaling axis. The immunohistochemical analysis demonstrating increasing expression of **A.** mCXCR2 **B.** mCXCL1 (*Continued*)

C

mCXCL3

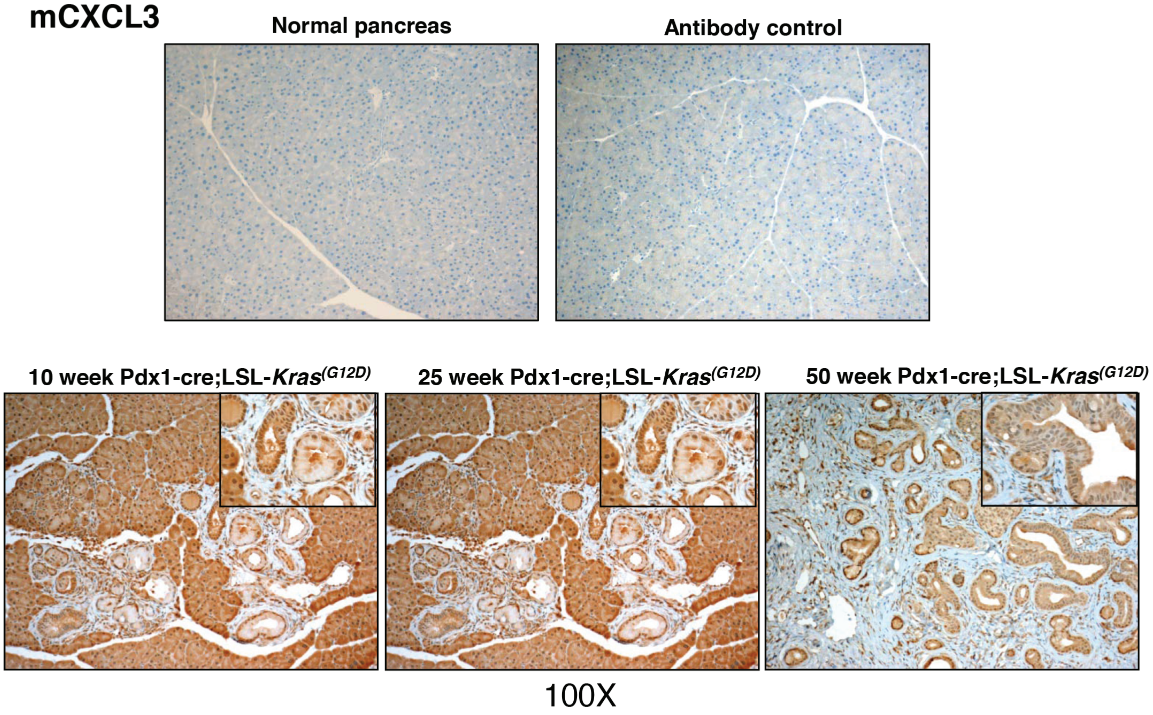

D

mCXCL5

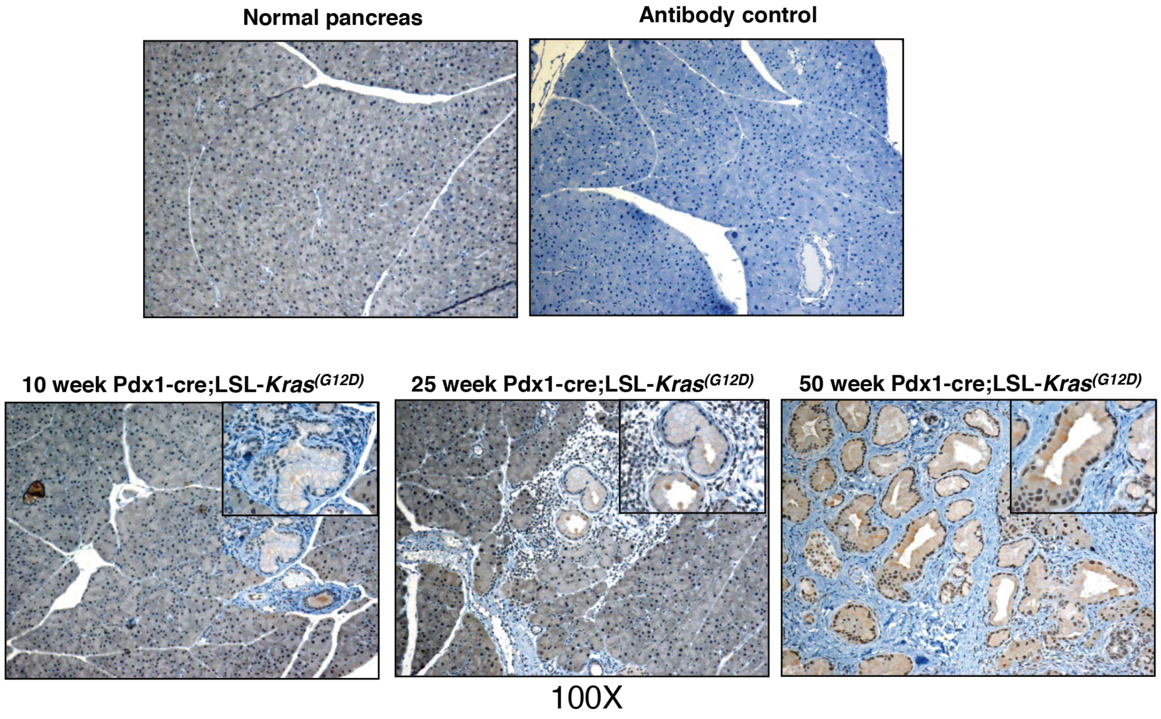

Supplementary Figure S2:(*Continued*) Lesions from Pdx1-cre;LSL-*Kras*$^{(G12D)}$ mice showing a progressive increase of CXCR2 signaling axis. C. mCXCL3 and **D.** mCXCL5 in developing lesions of Pdx1-cre;LSL-*Kras*$^{(G12D)}$ mice. Images in inset represent the higher magnification (400X) (*Continued*).

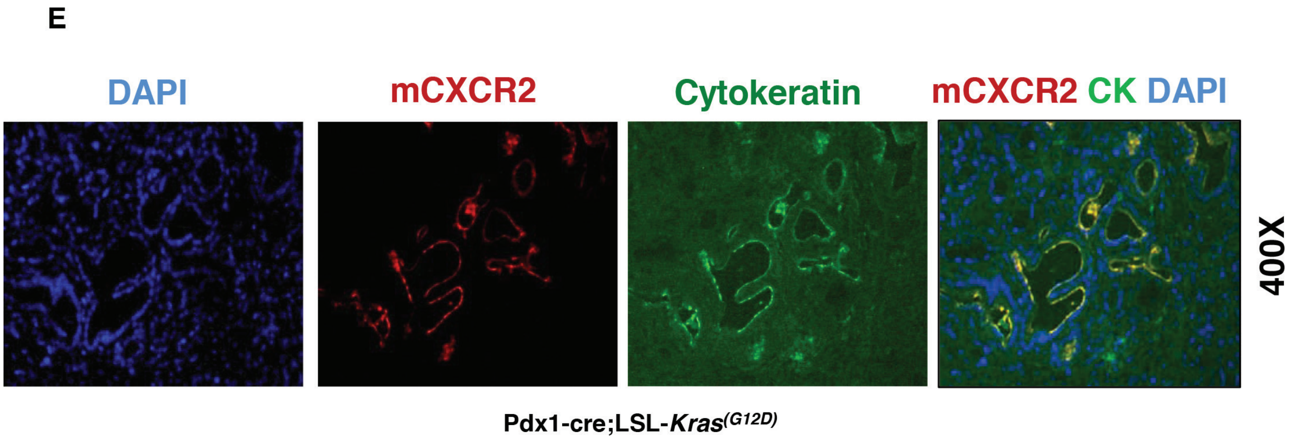

Supplementary Figure S2: (*Continued*) Lesions from Pdx1-cre;LSL-*Kras*$^{(G12D)}$ mice showing a progressive increase of CXCR2 signaling axis. E. Dualimmunofluorescence staining is demonstrating co-localization of mCXCR2 (Cy3) and cytokeratin (FITC) on the ductal cells. Nuclei arecounterstained with DAPI.

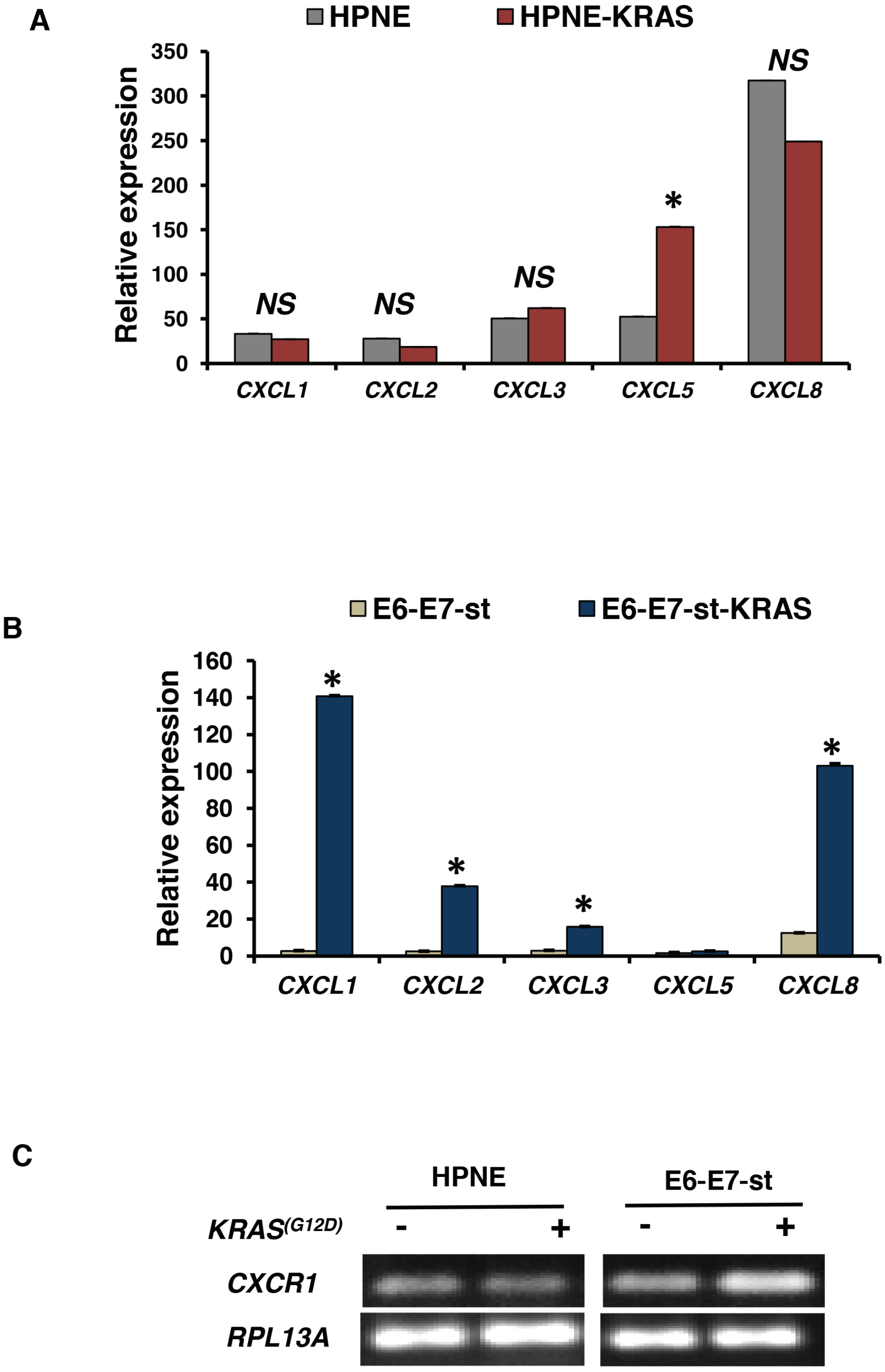

Supplementary Figure S3: Detection of RNA transcripts of ligands for CXCR2 in the *KRAS*$^{(G12D)}$- bearing pancreatic ductal cells. Real-time PCR for the detection of ligands for CXCR2 in **A.** HPNE/-KRAS and **B.** E6-E7-st/-KRAS cell models. **C.** Detection of transcripts for *CXCR1* in HPNE/-KRAS and E6-E7-st/-KRAS cell models. Statistical significance determined by paired Student's t-test (*$p \leq 0.05$, **$p \leq 0.01$, ***$p \leq 0.001$, *NS* $p > 0.05$).

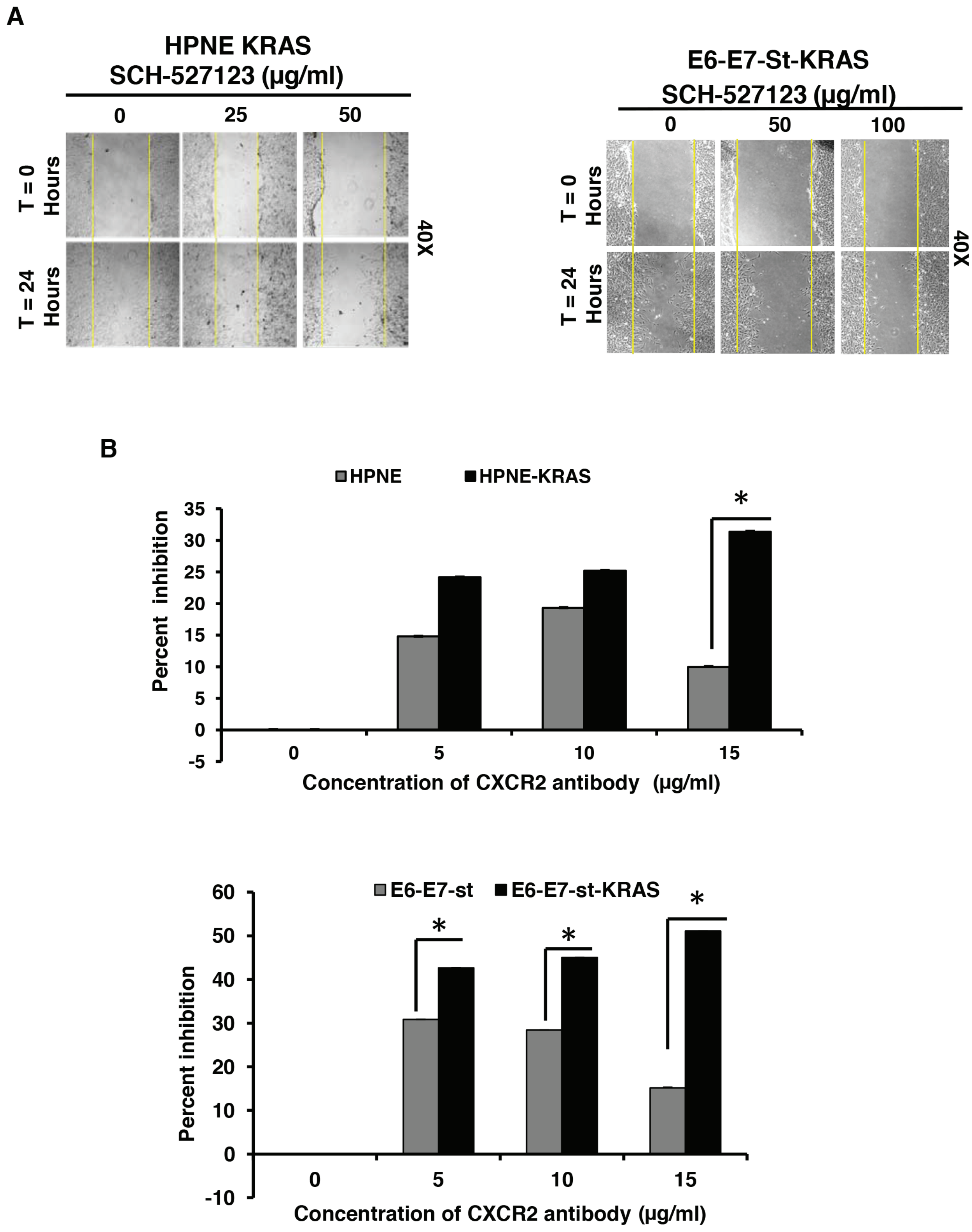

Supplementary Figure S4: Effect of inhibiting CXCR2 signaling on the *in vitro* phenotypes of the *KRAS*$^{(G12D)}$-bearing cells. A. Representative pictures of wound healing assay performed on HPNE-KRAS and E6-E7-st-KRAS cells by treating the cells with increasing doses of CXCR2 antagonist SCH-527123. **B.** Percent inhibition in cell viability by treatment with CXCR2 neutralizing antibody for 72 hours of HPNE/-KRAS and E6-E7-st/-KRAS cell models. Statistical significance determined by paired Student's t-test (*$p \leq 0.05$, **$p \leq 0.01$, ***$p \leq 0.001$, *NS* $p > 0.05$).

Supplementary Table S1: Details of the antibody used for the study

Species reactivity in this paper	Antibody	Supplier	Catalogue number	Host species	Dilution
**Immunohistochemistry and Immunofluorescence**					
Mouse	CXCR2	Kind gift from Dr. Strieter		Goat	1:1000
Mouse	Gro alpha	Abcam	ab86436	Rabbit	1:500
Mouse	CXCL3	Bioss	bs-2547R	Rabbit	1:500
Mouse	CXCL5	Cloud-clone corp.	PAA860Mu01	Rabbit	1:100
Human	Ki-67	Santacruz	sc-15402	Rabbit	1:50
Human	Cleaved caspase 3	Cell Signaling	Asp175	Rabbit	1:200
Mouse	Cytokeratin	Dako	Z0622	Rabbit	1:500
**Western blotting**					
Human	CXCR2	Santacruz	Sc-7304	Mouse	1:200
Human	ERK	Santacruz	Sc-94	Rabbit	1:200
Human	p-ERK	Cell Signaling	#9101	Rabbit	1:2000
Human	KRAS	Santacruz	F234-sc-30	Mouse	1:1000
Human	Actin	Sigma	A2066	Rabbit	1:5000

Supplementary Table S2: Details of the primers used for the study

mRNA	Temperature	In this study used for	Primer
Human Primers			
CXCR2	59°	Real-time PCR	Forward-5′-ACT TTT CCG AAG GAC CGT CT-3′<br>Reverse-5′- GTA ACA GCA TCC GCC AGT TT-3′
CXCR1	59°	Real-time PCR	Forward-5′-GAGCCCCGAATCTGACATTA-3′<br>Reverse-5′- GCAGACACTGCAACACACCT-3′
CXCL1	59°	Real-time PCR	Forward- 5′-ATT CAC CCC AAG AAC ATC CA-3′<br>Reverse- 5′-CAC CAG TGA GCT TCC TCC TC-3′
CXCL2	59°	Real-time PCR	Forward- 5′- GCA GGG AAT TCA CCT CAA GA-3′<br>Reverse- 5′- AGC TTC CTC CTT CCT TCT GG-3′
CXCL3	59°	Real-time PCR	Forward-5′- GCA GGG AAT TCA CCT CAA GA-3′<br>Reverse-5′- GGT GCT CCC CTT GTT CAG TA-3′
CXCL5	59°	Real-time PCR	Forward-5′- TCT GCA AGT GTT CGC CAT AG-3′<br>Reverse-5′-TTG TTT CCA CCG TCC AAA AT-3′
CXCL8	59°	Real-time PCR	Forward-5′- ACA TAC TCC AAA CCT TTC CAC-3′<br>Reverse-5′-CAA CCC TCT GCA CCC AGT TTT C-3′
RPL13A	59°	Real-time PCR and regular PCR	Forward-5′- GGCTGAAGCCTACCAGAAAG-3′<br>Reverse- 5′-CTTTGCCTTTTCCTTCCGTT-3
Murine Primers			
Cxcr2	60°	Regular PCR	Forward 5′- CACCGATGTCTACCTGCTGA -3′<br>Reverse 5′- CACAGGGTTGAGCCAAAAGT -3′
Cxcl1	55°	Regular PCR	Forward 5′-TCGCTTCTCTGTGCAGCGCT-3′<br>Reverse 5′- GTGGTTGACACTTAGTGGTCT C-3′
Cxcl2	57°	Regular PCR	Forward 5′-AGTGAACTGCGCTGTCAATG-3′<br>Reverse 5′-TTCAGGGTCAAGGCAAACTT-3′
Cxcl3	68°	Regular PCR	Forward 5′-GCAAGTCCAGCTGAGCCGGGA-3′<br>Reverse 5′-GACACCGTTGGGATGGATCGCTTT-3′
Cxcl5	68°	Regular PCR	Forward 5′-ATGGCGCCGCTGGCATTTCT-3′<br>Reverse 5′-CGCAGCTCCGTTGCGGCTAT-3′
Cxcl7	57°	Regular PCR	Forward 5′-CTCAGACCTTACATCGTCCTGC-3′<br>Reverse 5′-AGCGCAACAAGGATCGTCCTGC-3′
